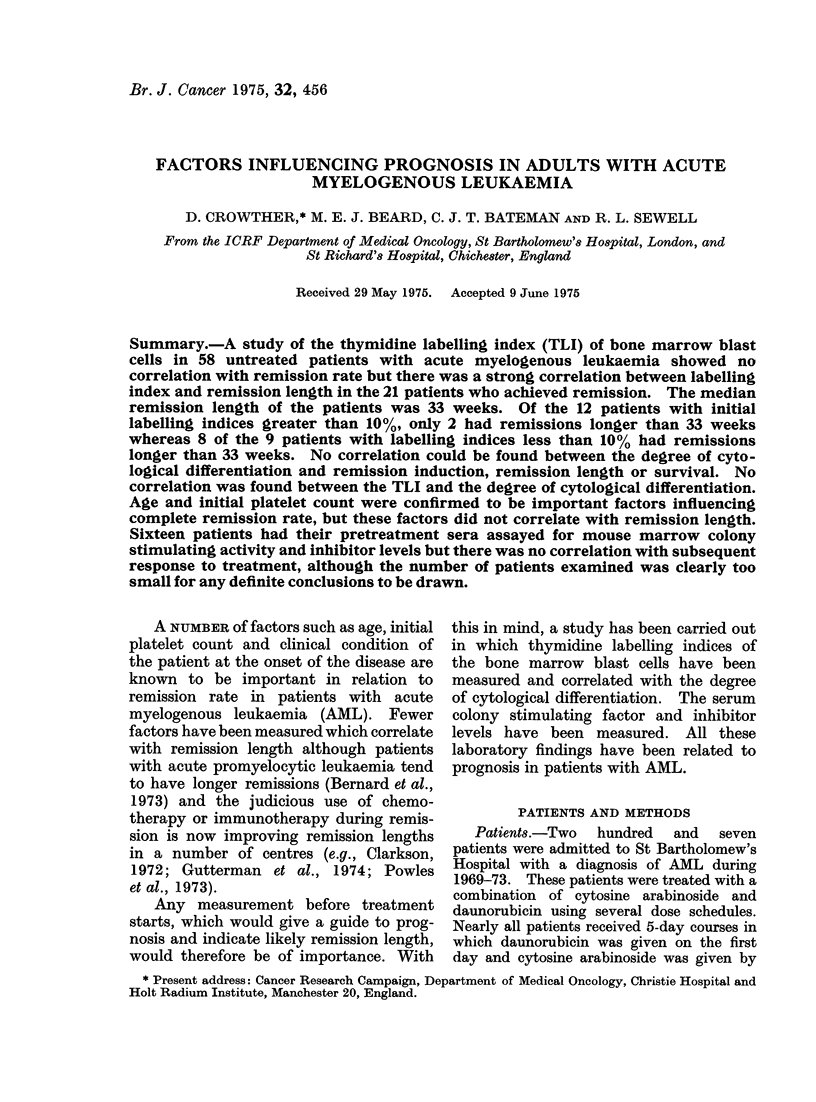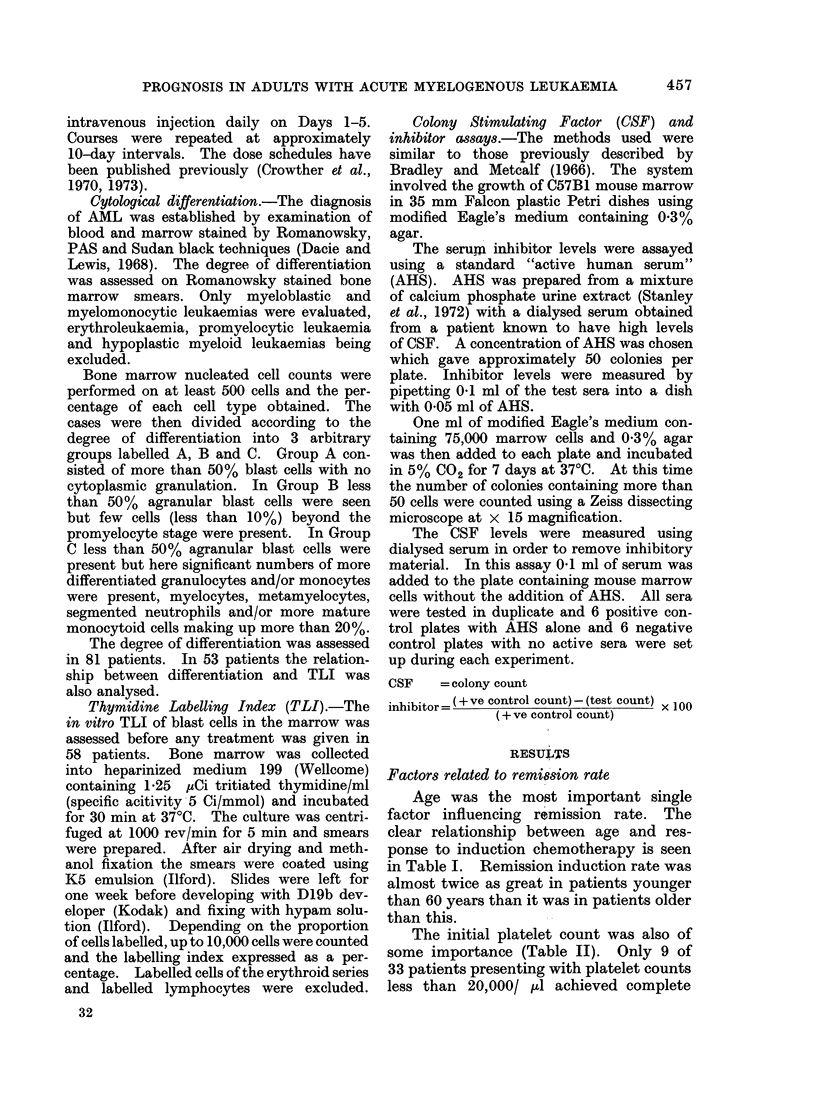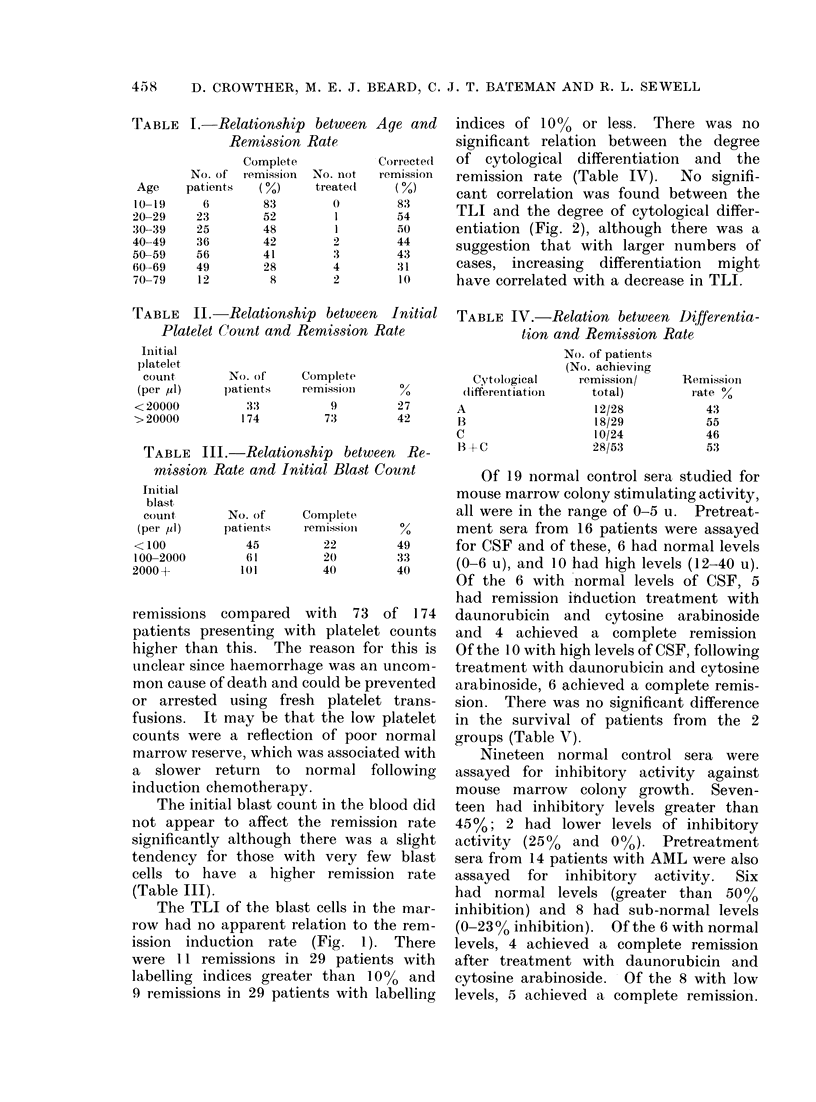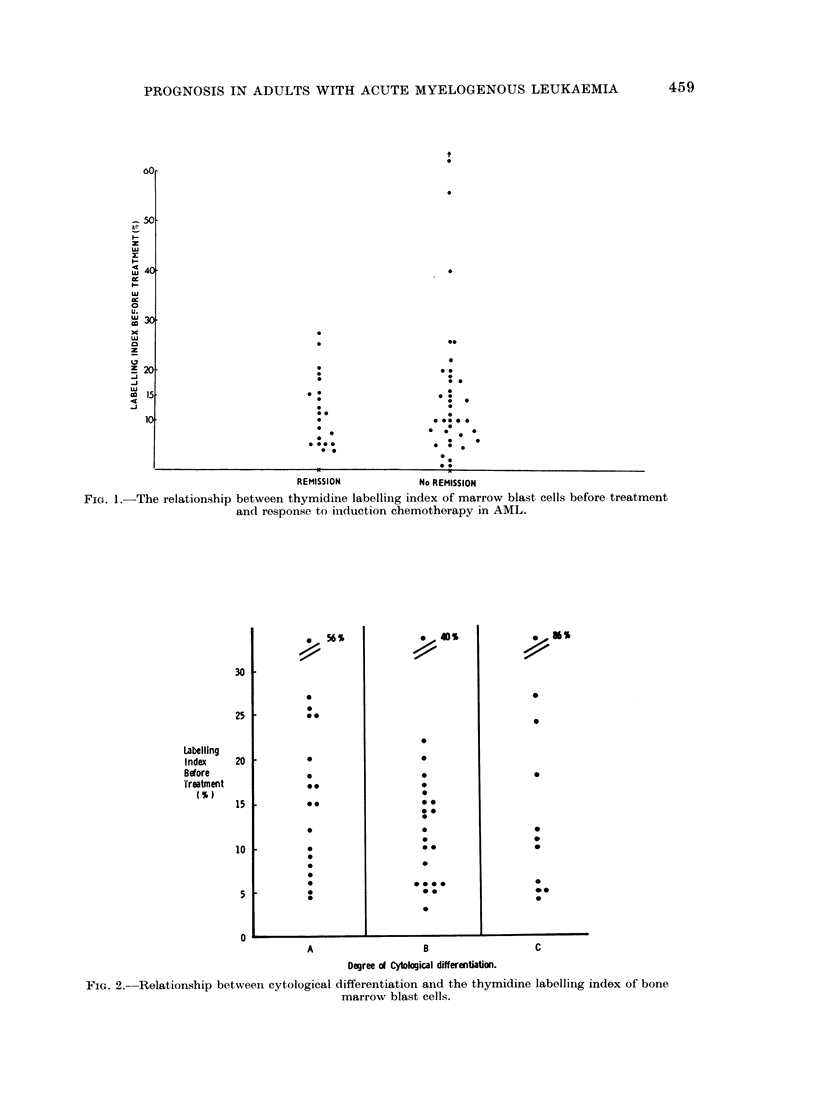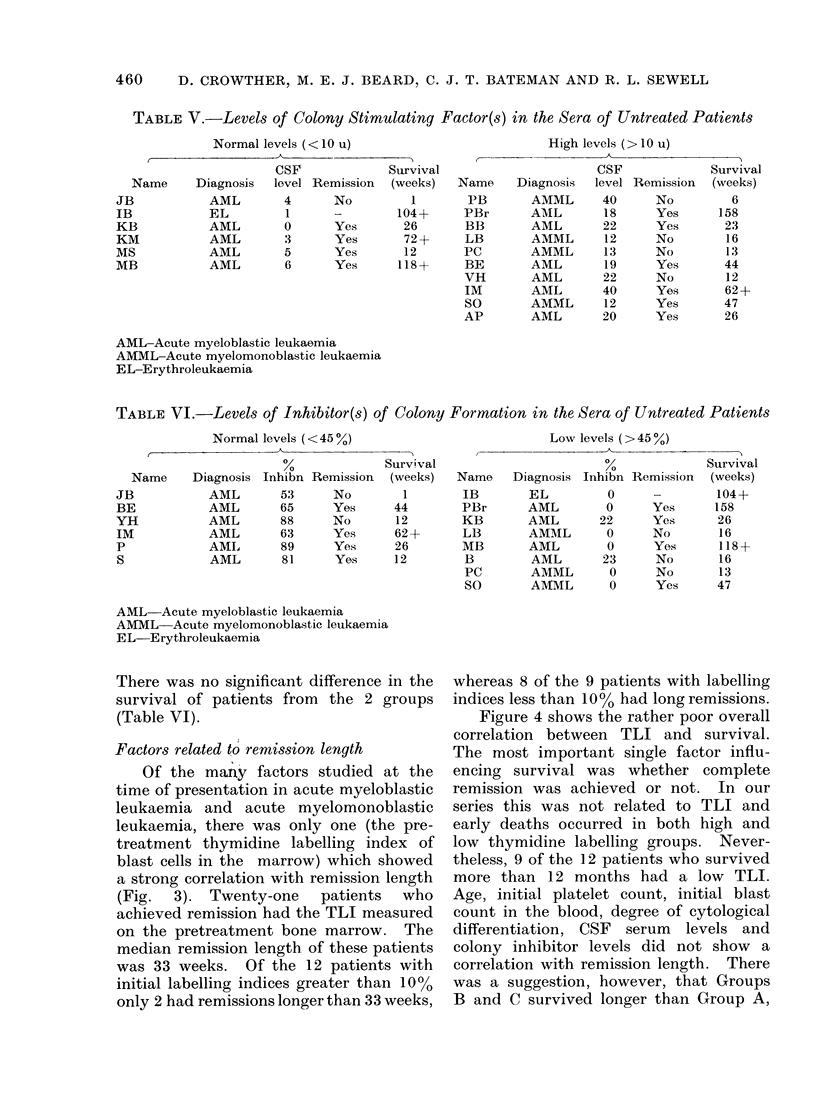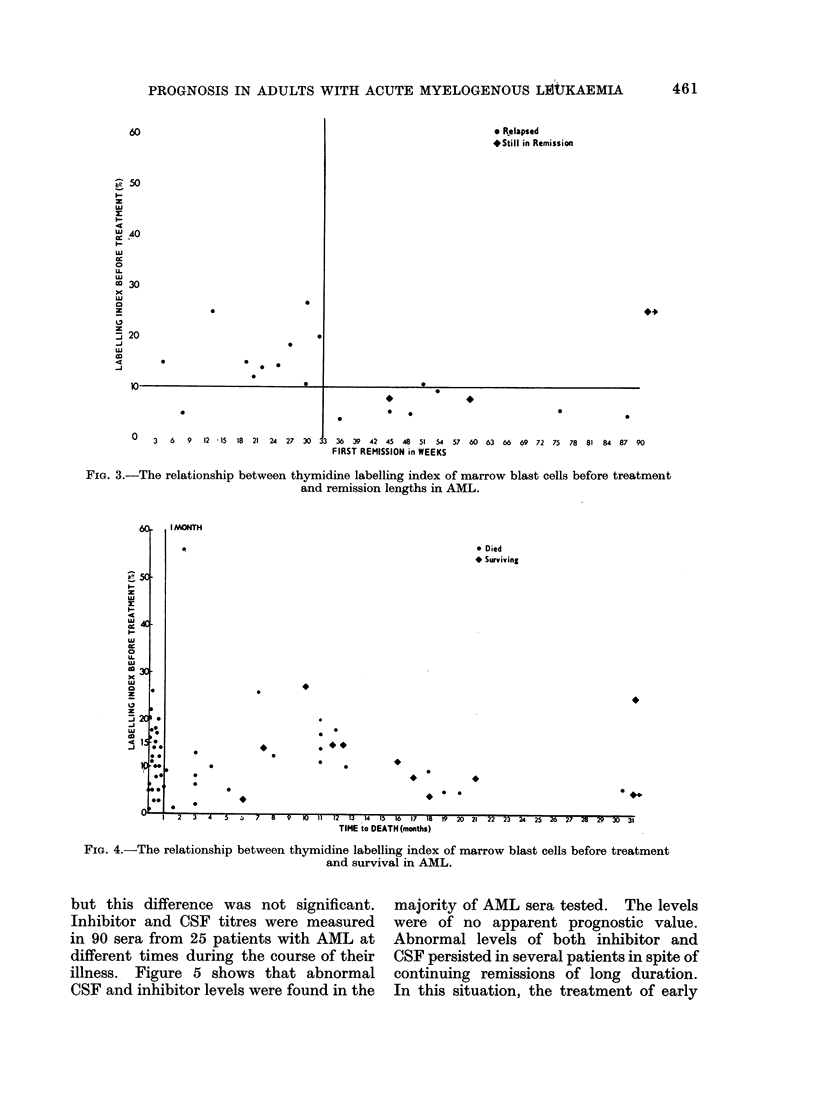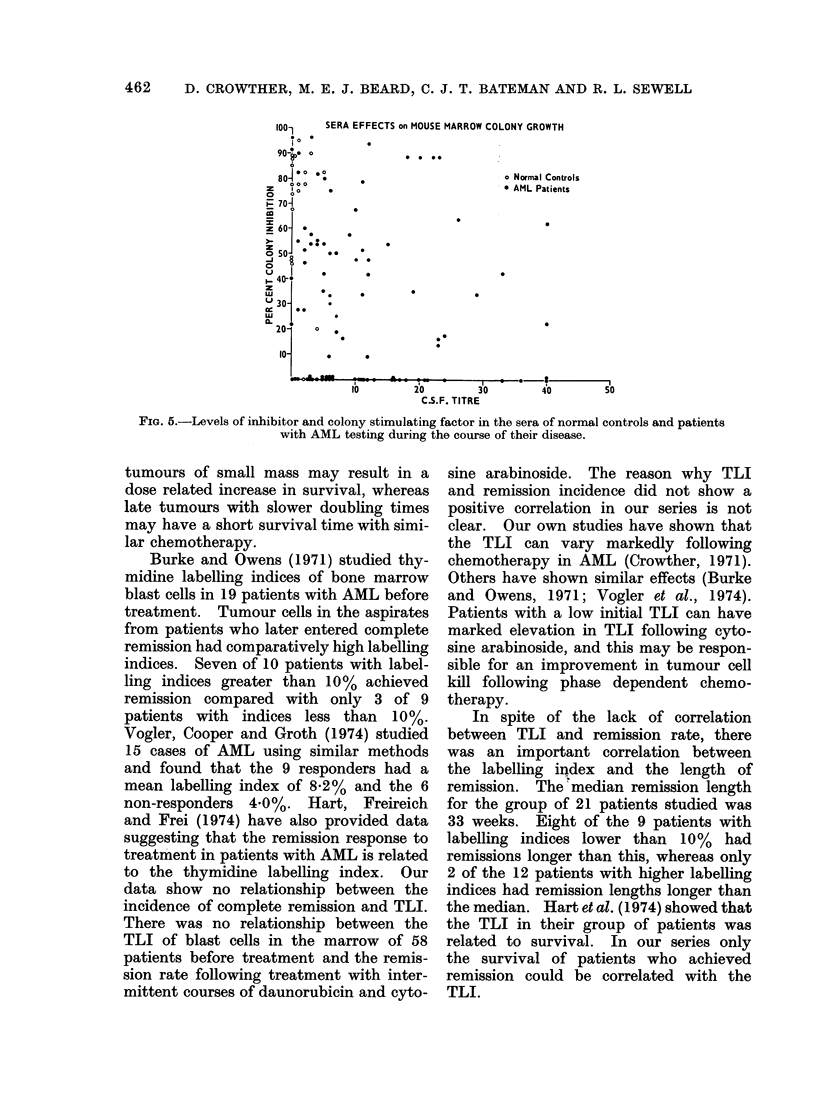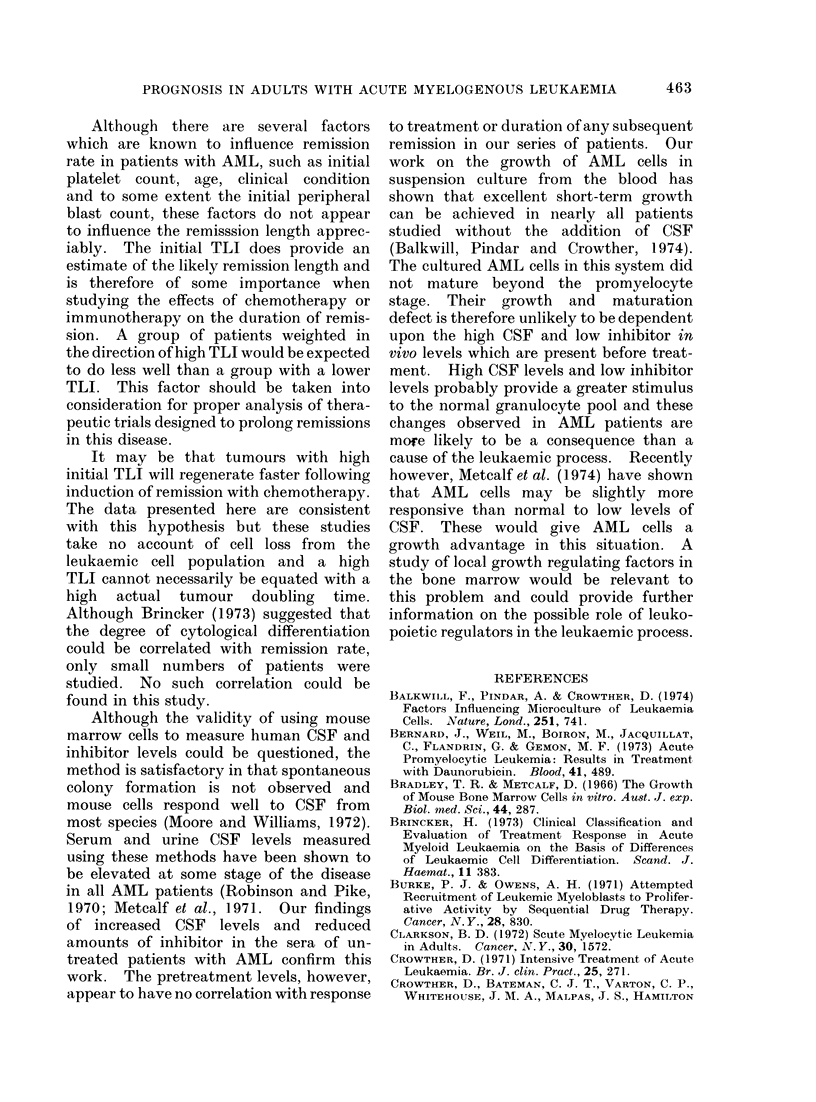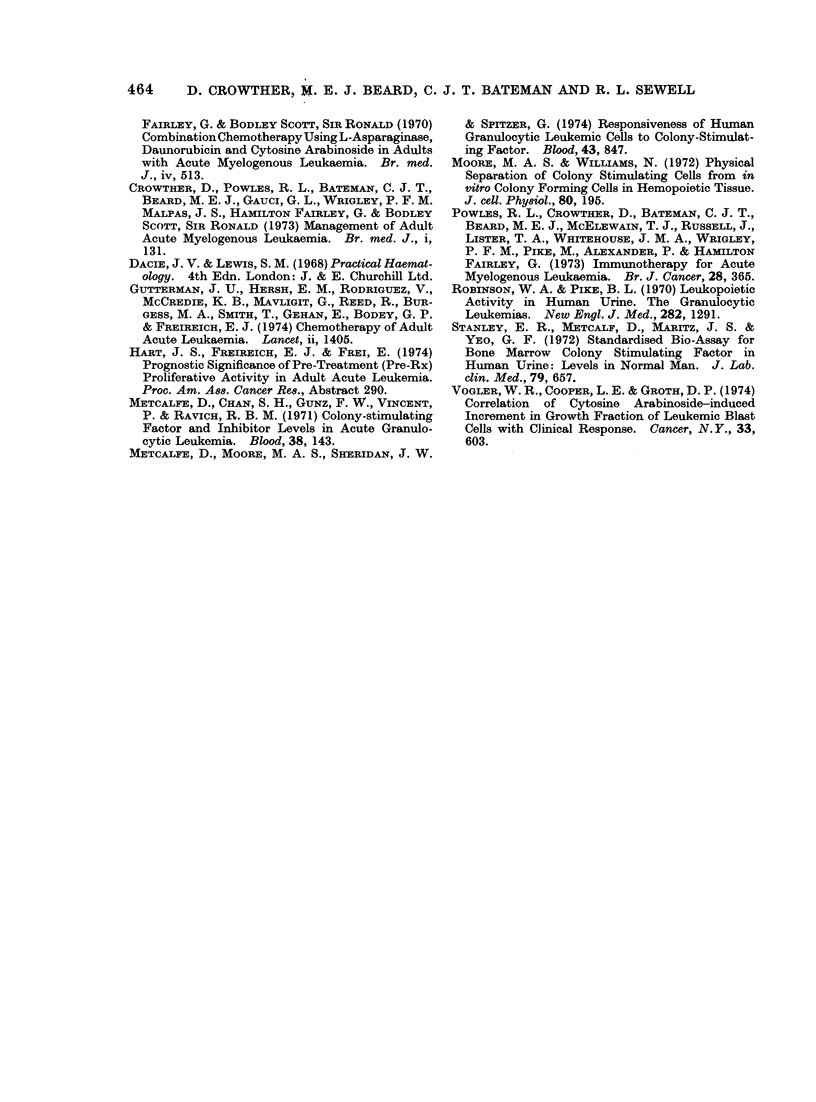# Factors influencing prognosis in adults with acute myelogenous leukaemia.

**DOI:** 10.1038/bjc.1975.247

**Published:** 1975-10

**Authors:** D. Crowther, M. E. Beard, C. J. Bateman, R. L. Sewell

## Abstract

A study of the thymidine labelling index (TLI) of bone marrow blast cells in 58 untreated patients with acute myelogenous leukemia showed no correlation with remission rate but there was a strong correlation between labelling index and remission length in the 21 patients who achieved remission. The median remission length of the patients was 33 weeks. Of the 12 patients with initial labelling indices greater than 10%, only 2 had remissions longer than 33 weeks whereas 8 of the 9 patients with labelling indices less than 10% had remissions longer than 33 weeks. No correlation could be found between the degree of cytological differentiation and remission induction, remission length or survival. No correlation was found between the TLI and the degree of cytological differentiation. Age and initial platelet count were confirmed to be important factors influencing complete remission rate, but these factors did not correlate with remission length. Sixteen patients had their pretreatment sera assayed for mouse marrow colony stimulating activity and inhibitor levels but there was no correlation with subsequent response to treatment, although the number of patients examined was clearly too small for any definite conclusions to be drawn.


*Br. J. Cancer* 1975, **32**, 456

### FACTORS INFLUENCING PROGNOSIS IN ADULTS WITH ACUTE MYELOGENOUS LEUKAEMIA

D. CROWTHER,* M. E. J. BEARD, C. J. T. BATEMAN AND R. L. SEWELL

*From the ICRF Department of Medical Oncology, St Bartholomew's Hospital, London, and St Richard's Hospital, Chichester, England*

Received 29 May 1975. Accepted 9 June 1975

**Summary.—A study of the thymidine labelling index (TLI) of bone marrow blast cells in 58 untreated patients with acute myelogenous leukaemia showed no correlation with remission rate but there was a strong correlation between labelling index and remission length in the 21 patients who achieved remission. The median remission length of the patients was 33 weeks. Of the 12 patients with initial labelling indices greater than 10%, only 2 had remissions longer than 33 weeks whereas 8 of the 9 patients with labelling indices less than 10% had remissions longer than 33 weeks. No correlation could be found between the degree of cytological differentiation and remission induction, remission length or survival. No correlation was found between the TLI and the degree of cytological differentiation. Age and initial platelet count were confirmed to be important factors influencing complete remission rate, but these factors did not correlate with remission length. Sixteen patients had their pretreatment sera assayed for mouse marrow colony stimulating activity and inhibitor levels but there was no correlation with subsequent response to treatment, although the number of patients examined was clearly too small for any definite conclusions to be drawn.**

A NUMBER of factors such as age, initial platelet count and clinical condition of the patient at the onset of the disease are known to be important in relation to remission rate in patients with acute myelogenous leukaemia (AML). Fewer factors have been measured which correlate with remission length although patients with acute promyelocytic leukaemia tend to have longer remissions (Bernard *et al.*, 1973) and the judicious use of chemotherapy or immunotherapy during remission is now improving remission lengths in a number of centres (*e.g.*, Clarkson, 1972; Gutterman *et al.*, 1974; Powles *et al.*, 1973).

Any measurement before treatment starts, which would give a guide to prognosis and indicate likely remission length, would therefore be of importance. With this in mind, a study has been carried out in which thymidine labelling indices of the bone marrow blast cells have been measured and correlated with the degree of cytological differentiation. The serum colony stimulating factor and inhibitor levels have been measured. All these laboratory findings have been related to prognosis in patients with AML.

### PATIENTS AND METHODS

*Patients.*—Two hundred and seven patients were admitted to St Bartholomew's Hospital with a diagnosis of AML during 1969–73. These patients were treated with a combination of cytosine arabinoside and daunorubicin using several dose schedules. Nearly all patients received 5-day courses in which daunorubicin was given on the first day and cytosine arabinoside was given by

* Present address: Cancer Research Campaign, Department of Medical Oncology, Christie Hospital and Holt Radium Institute, Manchester 20, England.

intravenous injection daily on Days 1–5. Courses were repeated at approximately 10–day intervals. The dose schedules have been published previously (Crowther *et al.*, 1970, 1973).

*Cytological differentiation.*—The diagnosis of AML was established by examination of blood and marrow stained by Romanowsky, PAS and Sudan black techniques (Dacie and Lewis, 1968). The degree of differentiation was assessed on Romanowsky stained bone marrow smears. Only myeloblastic and myelomonocytic leukaemias were evaluated, erythroleukaemia, promyelocytic leukaemia and hypoplastic myeloid leukaemias being excluded.

Bone marrow nucleated cell counts were performed on at least 500 cells and the percentage of each cell type obtained. The cases were then divided according to the degree of differentiation into 3 arbitrary groups labelled A, B and C. Group A consisted of more than 50% blast cells with no cytoplasmic granulation. In Group B less than 50% agranular blast cells were seen but few cells (less than 10%) beyond the promyelocyte stage were present. In Group C less than 50% agranular blast cells were present but here significant numbers of more differentiated granulocytes and/or monocytes were present, myelocytes, metamyelocytes, segmented neutrophils and/or more mature monocytoid cells making up more than 20%.

The degree of differentiation was assessed in 81 patients. In 53 patients the relationship between differentiation and TLI was also analysed.

*Thymidine Labelling Index (TLI).*—The *in vitro* TLI of blast cells in the marrow was assessed before any treatment was given in 58 patients. Bone marrow was collected into heparinized medium 199 (Wellcome) containing 1·25 μCi tritiated thymidine/ml (specific acitivity 5 Ci/mmol) and incubated for 30 min at 37°C. The culture was centrifuged at 1000 rev/min for 5 min and smears were prepared. After air drying and methanol fixation the smears were coated using K5 emulsion (Ilford). Slides were left for one week before developing with D19b developer (Kodak) and fixing with hypam solution (Ilford). Depending on the proportion of cells labelled, up to 10,000 cells were counted and the labelling index expressed as a percentage. Labelled cells of the erythroid series and labelled lymphocytes were excluded.

*Colony Stimulating Factor (CSF) and inhibitor assays.*—The methods used were similar to those previously described by Bradley and Metcalf (1966). The system involved the growth of C57Bl mouse marrow in 35 mm Falcon plastic Petri dishes using modified Eagle's medium containing 0·3% agar.

The serum inhibitor levels were assayed using a standard "active human serum" (AHS). AHS was prepared from a mixture of calcium phosphate urine extract (Stanley *et al.*, 1972) with a dialysed serum obtained from a patient known to have high levels of CSF. A concentration of AHS was chosen which gave approximately 50 colonies per plate. Inhibitor levels were measured by pipetting 0·1 ml of the test sera into a dish with 0·05 ml of AHS.

One ml of modified Eagle's medium containing 75,000 marrow cells and 0·3% agar was then added to each plate and incubated in 5% $CO_2$ for 7 days at 37°C. At this time the number of colonies containing more than 50 cells were counted using a Zeiss dissecting microscope at × 15 magnification.

The CSF levels were measured using dialysed serum in order to remove inhibitory material. In this assay 0·1 ml of serum was added to the plate containing mouse marrow cells without the addition of AHS. All sera were tested in duplicate and 6 positive control plates with AHS alone and 6 negative control plates with no active sera were set up during each experiment.

CSF = colony count

$$\text{inhibitor} = \frac{(+\text{ve control count}) - (\text{test count})}{(+\text{ve control count})} \times 100$$

### RESULTS

### *Factors related to remission rate*

Age was the most important single factor influencing remission rate. The clear relationship between age and response to induction chemotherapy is seen in Table I. Remission induction rate was almost twice as great in patients younger than 60 years than it was in patients older than this.

The initial platelet count was also of some importance (Table II). Only 9 of 33 patients presenting with platelet counts less than 20,000/ μl achieved complete

TABLE I.—*Relationship between Age and Remission Rate*

| Age | No. of patients | Complete remission (%) | No. not treated | Corrected remission (%) |
|---|---|---|---|---|
| 10–19 | 6 | 83 | 0 | 83 |
| 20–29 | 23 | 52 | 1 | 54 |
| 30–39 | 25 | 48 | 1 | 50 |
| 40–49 | 36 | 42 | 2 | 44 |
| 50–59 | 56 | 41 | 3 | 43 |
| 60–69 | 49 | 28 | 4 | 31 |
| 70–79 | 12 | 8 | 2 | 10 |

TABLE II.—*Relationship between Initial Platelet Count and Remission Rate*

| Initial platelet count (per μl) | No. of patients | Complete remission | % |
|---|---|---|---|
| <20000 | 33 | 9 | 27 |
| >20000 | 174 | 73 | 42 |

TABLE III.—*Relationship between Remission Rate and Initial Blast Count*

| Initial blast count (per μl) | No. of patients | Complete remission | % |
|---|---|---|---|
| <100 | 45 | 22 | 49 |
| 100–2000 | 61 | 20 | 33 |
| 2000+ | 101 | 40 | 40 |

remissions compared with 73 of 174 patients presenting with platelet counts higher than this. The reason for this is unclear since haemorrhage was an uncommon cause of death and could be prevented or arrested using fresh platelet transfusions. It may be that the low platelet counts were a reflection of poor normal marrow reserve, which was associated with a slower return to normal following induction chemotherapy.

The initial blast count in the blood did not appear to affect the remission rate significantly although there was a slight tendency for those with very few blast cells to have a higher remission rate (Table III).

The TLI of the blast cells in the marrow had no apparent relation to the remission induction rate (Fig. 1). There were 11 remissions in 29 patients with labelling indices greater than 10% and 9 remissions in 29 patients with labelling indices of 10% or less. There was no significant relation between the degree of cytological differentiation and the remission rate (Table IV). No significant correlation was found between the TLI and the degree of cytological differentiation (Fig. 2), although there was a suggestion that with larger numbers of cases, increasing differentiation might have correlated with a decrease in TLI.

TABLE IV.—*Relation between Differentiation and Remission Rate*

| Cytological differentiation | No. of patients (No. achieving remission/ total) | Remission rate % |
|---|---|---|
| A | 12/28 | 43 |
| B | 18/29 | 55 |
| C | 10/24 | 46 |
| B+C | 28/53 | 53 |

Of 19 normal control sera studied for mouse marrow colony stimulating activity, all were in the range of 0–5 u. Pretreatment sera from 16 patients were assayed for CSF and of these, 6 had normal levels (0–6 u), and 10 had high levels (12–40 u). Of the 6 with normal levels of CSF, 5 had remission induction treatment with daunorubicin and cytosine arabinoside and 4 achieved a complete remission Of the 10 with high levels of CSF, following treatment with daunorubicin and cytosine arabinoside, 6 achieved a complete remission. There was no significant difference in the survival of patients from the 2 groups (Table V).

Nineteen normal control sera were assayed for inhibitory activity against mouse marrow colony growth. Seventeen had inhibitory levels greater than 45%; 2 had lower levels of inhibitory activity (25% and 0%). Pretreatment sera from 14 patients with AML were also assayed for inhibitory activity. Six had normal levels (greater than 50% inhibition) and 8 had sub-normal levels (0–23% inhibition). Of the 6 with normal levels, 4 achieved a complete remission after treatment with daunorubicin and cytosine arabinoside. Of the 8 with low levels, 5 achieved a complete remission.

FIG. 1.—The relationship between thymidine labelling index of marrow blast cells before treatment and response to induction chemotherapy in AML.

FIG. 2.—Relationship between cytological differentiation and the thymidine labelling index of bone marrow blast cells.

TABLE V.—*Levels of Colony Stimulating Factor(s) in the Sera of Untreated Patients*

| Normal levels (<10 u) | | | | | High levels (>10 u) | | | | |
|---|---|---|---|---|---|---|---|---|---|
| Name | Diagnosis | CSF level | Remission | Survival (weeks) | Name | Diagnosis | CSF level | Remission | Survival (weeks) |
| JB | AML | 4 | No | 1 | PB | AMML | 40 | No | 6 |
| IB | EL | 1 | - | 104+ | PBr | AML | 18 | Yes | 158 |
| KB | AML | 0 | Yes | 26 | BB | AML | 22 | Yes | 23 |
| KM | AML | 3 | Yes | 72+ | LB | AMML | 12 | No | 16 |
| MS | AML | 5 | Yes | 12 | PC | AMML | 13 | No | 13 |
| MB | AML | 6 | Yes | 118+ | BE | AML | 19 | Yes | 44 |
| | | | | | VH | AML | 22 | No | 12 |
| | | | | | IM | AML | 40 | Yes | 62+ |
| | | | | | SO | AMML | 12 | Yes | 47 |
| | | | | | AP | AML | 20 | Yes | 26 |

AML–Acute myeloblastic leukaemia
AMML–Acute myelomonoblastic leukaemia
EL–Erythroleukaemia

TABLE VI.—*Levels of Inhibitor(s) of Colony Formation in the Sera of Untreated Patients*

| Normal levels (<45%) | | | | | Low levels (>45%) | | | | |
|---|---|---|---|---|---|---|---|---|---|
| Name | Diagnosis | % Inhibn | Remission | Survival (weeks) | Name | Diagnosis | % Inhibn | Remission | Survival (weeks) |
| JB | AML | 53 | No | 1 | IB | EL | 0 | - | 104+ |
| BE | AML | 65 | Yes | 44 | PBr | AML | 0 | Yes | 158 |
| YH | AML | 88 | No | 12 | KB | AML | 22 | Yes | 26 |
| IM | AML | 63 | Yes | 62+ | LB | AMML | 0 | No | 16 |
| P | AML | 89 | Yes | 26 | MB | AML | 0 | Yes | 118+ |
| S | AML | 81 | Yes | 12 | B | AML | 23 | No | 16 |
| | | | | | PC | AMML | 0 | No | 13 |
| | | | | | SO | AMML | 0 | Yes | 47 |

AML—Acute myeloblastic leukaemia
AMML—Acute myelomonoblastic leukaemia
EL—Erythroleukaemia

There was no significant difference in the survival of patients from the 2 groups (Table VI).

*Factors related to remission length*

Of the many factors studied at the time of presentation in acute myeloblastic leukaemia and acute myelomonoblastic leukaemia, there was only one (the pretreatment thymidine labelling index of blast cells in the marrow) which showed a strong correlation with remission length (Fig. 3). Twenty-one patients who achieved remission had the TLI measured on the pretreatment bone marrow. The median remission length of these patients was 33 weeks. Of the 12 patients with initial labelling indices greater than 10% only 2 had remissions longer than 33 weeks, whereas 8 of the 9 patients with labelling indices less than 10% had long remissions.

Figure 4 shows the rather poor overall correlation between TLI and survival. The most important single factor influencing survival was whether complete remission was achieved or not. In our series this was not related to TLI and early deaths occurred in both high and low thymidine labelling groups. Nevertheless, 9 of the 12 patients who survived more than 12 months had a low TLI. Age, initial platelet count, initial blast count in the blood, degree of cytological differentiation, CSF serum levels and colony inhibitor levels did not show a correlation with remission length. There was a suggestion, however, that Groups B and C survived longer than Group A,

FIG. 3.—The relationship between thymidine labelling index of marrow blast cells before treatment and remission lengths in AML.

FIG. 4.—The relationship between thymidine labelling index of marrow blast cells before treatment and survival in AML.

but this difference was not significant. Inhibitor and CSF titres were measured in 90 sera from 25 patients with AML at different times during the course of their illness. Figure 5 shows that abnormal CSF and inhibitor levels were found in the majority of AML sera tested. The levels were of no apparent prognostic value. Abnormal levels of both inhibitor and CSF persisted in several patients in spite of continuing remissions of long duration. In this situation, the treatment of early

FIG. 5.—Levels of inhibitor and colony stimulating factor in the sera of normal controls and patients with AML testing during the course of their disease.

tumours of small mass may result in a dose related increase in survival, whereas late tumours with slower doubling times may have a short survival time with similar chemotherapy.

Burke and Owens (1971) studied thymidine labelling indices of bone marrow blast cells in 19 patients with AML before treatment. Tumour cells in the aspirates from patients who later entered complete remission had comparatively high labelling indices. Seven of 10 patients with labelling indices greater than 10% achieved remission compared with only 3 of 9 patients with indices less than 10%. Vogler, Cooper and Groth (1974) studied 15 cases of AML using similar methods and found that the 9 responders had a mean labelling index of 8·2% and the 6 non-responders 4·0%. Hart, Freireich and Frei (1974) have also provided data suggesting that the remission response to treatment in patients with AML is related to the thymidine labelling index. Our data show no relationship between the incidence of complete remission and TLI. There was no relationship between the TLI of blast cells in the marrow of 58 patients before treatment and the remission rate following treatment with intermittent courses of daunorubicin and cytosine arabinoside. The reason why TLI and remission incidence did not show a positive correlation in our series is not clear. Our own studies have shown that the TLI can vary markedly following chemotherapy in AML (Crowther, 1971). Others have shown similar effects (Burke and Owens, 1971; Vogler *et al.*, 1974). Patients with a low initial TLI can have marked elevation in TLI following cytosine arabinoside, and this may be responsible for an improvement in tumour cell kill following phase dependent chemotherapy.

In spite of the lack of correlation between TLI and remission rate, there was an important correlation between the labelling index and the length of remission. The median remission length for the group of 21 patients studied was 33 weeks. Eight of the 9 patients with labelling indices lower than 10% had remissions longer than this, whereas only 2 of the 12 patients with higher labelling indices had remission lengths longer than the median. Hart *et al.* (1974) showed that the TLI in their group of patients was related to survival. In our series only the survival of patients who achieved remission could be correlated with the TLI.

Although there are several factors which are known to influence remission rate in patients with AML, such as initial platelet count, age, clinical condition and to some extent the initial peripheral blast count, these factors do not appear to influence the remisssion length appreciably. The initial TLI does provide an estimate of the likely remission length and is therefore of some importance when studying the effects of chemotherapy or immunotherapy on the duration of remission. A group of patients weighted in the direction of high TLI would be expected to do less well than a group with a lower TLI. This factor should be taken into consideration for proper analysis of therapeutic trials designed to prolong remissions in this disease.

It may be that tumours with high initial TLI will regenerate faster following induction of remission with chemotherapy. The data presented here are consistent with this hypothesis but these studies take no account of cell loss from the leukaemic cell population and a high TLI cannot necessarily be equated with a high actual tumour doubling time. Although Brincker (1973) suggested that the degree of cytological differentiation could be correlated with remission rate, only small numbers of patients were studied. No such correlation could be found in this study.

Although the validity of using mouse marrow cells to measure human CSF and inhibitor levels could be questioned, the method is satisfactory in that spontaneous colony formation is not observed and mouse cells respond well to CSF from most species (Moore and Williams, 1972). Serum and urine CSF levels measured using these methods have been shown to be elevated at some stage of the disease in all AML patients (Robinson and Pike, 1970; Metcalf *et al.*, 1971. Our findings of increased CSF levels and reduced amounts of inhibitor in the sera of untreated patients with AML confirm this work. The pretreatment levels, however, appear to have no correlation with response to treatment or duration of any subsequent remission in our series of patients. Our work on the growth of AML cells in suspension culture from the blood has shown that excellent short-term growth can be achieved in nearly all patients studied without the addition of CSF (Balkwill, Pindar and Crowther, 1974). The cultured AML cells in this system did not mature beyond the promyelocyte stage. Their growth and maturation defect is therefore unlikely to be dependent upon the high CSF and low inhibitor *in vivo* levels which are present before treatment. High CSF levels and low inhibitor levels probably provide a greater stimulus to the normal granulocyte pool and these changes observed in AML patients are more likely to be a consequence than a cause of the leukaemic process. Recently however, Metcalf *et al.* (1974) have shown that AML cells may be slightly more responsive than normal to low levels of CSF. These would give AML cells a growth advantage in this situation. A study of local growth regulating factors in the bone marrow would be relevant to this problem and could provide further information on the possible role of leukopoietic regulators in the leukaemic process.

### REFERENCES

Balkwill, F., Pindar, A. & Crowther, D. (1974) Factors Influencing Microculture of Leukaemia Cells. *Nature, Lond.*, **251**, 741.

Bernard, J., Weil, M., Boiron, M., Jacquillat, C., Flandrin, G. & Gemon, M. F. (1973) Acute Promyelocytic Leukemia: Results in Treatment with Daunorubicin. *Blood*, **41**, 489.

Bradley, T. R. & Metcalf, D. (1966) The Growth of Mouse Bone Marrow Cells *in vitro*. *Aust. J. exp. Biol. med. Sci.*, **44**, 287.

Brincker, H. (1973) Clinical Classification and Evaluation of Treatment Response in Acute Myeloid Leukaemia on the Basis of Differences of Leukaemic Cell Differentiation. *Scand. J. Haemat.*, **11** 383.

Burke, P. J. & Owens, A. H. (1971) Attempted Recruitment of Leukemic Myeloblasts to Proliferative Activity by Sequential Drug Therapy. *Cancer, N.Y.*, **28**, 830.

Clarkson, B. D. (1972) Scute Myelocytic Leukemia in Adults. *Cancer, N.Y.*, **30**, 1572.

Crowther, D. (1971) Intensive Treatment of Acute Leukaemia. *Br. J. clin. Pract.*, **25**, 271.

Crowther, D., Bateman, C. J. T., Varton, C. P., Whitehouse, J. M. A., Malpas, J. S., Hamilton

Fairley, G. & Bodley Scott, Sir Ronald (1970) Combination Chemotherapy Using L-Asparaginase, Daunorubicin and Cytosine Arabinoside in Adults with Acute Myelogenous Leukaemia. *Br. med. J.*, iv, 513.

Crowther, D., Powles, R. L., Bateman, C. J. T., Beard, M. E. J., Gauci, G. L., Wrigley, P. F. M. Malpas, J. S., Hamilton Fairley, G. & Bodley Scott, Sir Ronald (1973) Management of Adult Acute Myelogenous Leukaemia. *Br. med. J.*, i, 131.

Dacie, J. V. & Lewis, S. M. (1968) *Practical Haematology.* 4th Edn. London: J. & E. Churchill Ltd.

Gutterman, J. U., Hersh, E. M., Rodriguez, V., McCredie, K. B., Mavligit, G., Reed, R., Burgess, M. A., Smith, T., Gehan, E., Bodey, G. P. & Freireich, E. J. (1974) Chemotherapy of Adult Acute Leukaemia. *Lancet*, ii, 1405.

Hart, J. S., Freireich, E. J. & Frei, E. (1974) Prognostic Significance of Pre-Treatment (Pre-Rx) Proliferative Activity in Adult Acute Leukemia. *Proc. Am. Ass. Cancer Res.*, Abstract 290.

Metcalfe, D., Chan, S. H., Gunz, F. W., Vincent, P. & Ravich, R. B. M. (1971) Colony-stimulating Factor and Inhibitor Levels in Acute Granulocytic Leukemia. *Blood*, **38**, 143.

Metcalfe, D., Moore, M. A. S., Sheridan, J. W. & Spitzer, G. (1974) Responsiveness of Human Granulocytic Leukemic Cells to Colony-Stimulating Factor. *Blood*, **43**, 847.

Moore, M. A. S. & Williams, N. (1972) Physical Separation of Colony Stimulating Cells from *in vitro* Colony Forming Cells in Hemopoietic Tissue. *J. cell. Physiol.*, **80**, 195.

Powles, R. L., Crowther, D., Bateman, C. J. T., Beard, M. E. J., McElewain, T. J., Russell, J., Lister, T. A., Whitehouse, J. M. A., Wrigley, P. F. M., Pike, M., Alexander, P. & Hamilton Fairley, G. (1973) Immunotherapy for Acute Myelogenous Leukaemia. *Br. J. Cancer*, **28**, 365.

Robinson, W. A. & Pike, B. L. (1970) Leukopoietic Activity in Human Urine. The Granulocytic Leukemias. *New Engl. J. Med.*, **282**, 1291.

Stanley, E. R., Metcalf, D., Maritz, J. S. & Yeo, G. F. (1972) Standardised Bio-Assay for Bone Marrow Colony Stimulating Factor in Human Urine: Levels in Normal Man. *J. Lab. clin. Med.*, **79**, 657.

Vogler, W. R., Cooper, L. E. & Groth, D. P. (1974) Correlation of Cytosine Arabinoside–induced Increment in Growth Fraction of Leukemic Blast Cells with Clinical Response. *Cancer, N.Y.*, **33**, 603.